# Accuracy in Measurement of Infant Formula Powder and Water by Caregivers With and Without a Crying Baby Present

**DOI:** 10.1001/jamanetworkopen.2024.47362

**Published:** 2024-12-04

**Authors:** Richard R. Rosenkranz, Chris Acosta, Ana Gonzalez-Alvarez, Andrew Hooyman, Jose R. Hidalgo, Romina Ballesteros-Paniagua, Sara K. Rosenkranz

**Affiliations:** 1 Department of Kinesiology and Nutrition Sciences, School of Integrated Health Sciences, University of Nevada, Las Vegas; 2 Department of Social and Behavioral Health, School of Public Health, University of Nevada, Las Vegas

## Abstract

This cross-sectional study assesses the accuracy in measurement of powder and water when preparing infant formula in the presence or absence of a simulated crying baby.

## Introduction

Infant formula provides a safe, nutritious alternative to breastfeeding but caregivers may use unsanitary methods or make substantial errors with formula dilution and jeopardize infant health.^1^ Underdiluting formula can result in health problems, such as hypernatremic dehydration, gastroenteritis, other digestive problems, or long-term excess weight gain.^2^ Overdiluting infant formula, sometimes used to reduce expense by low-income caregivers,^2^ can also lead to serious health problems for babies, including diarrhea, water intoxication, nutrient deficiencies, malnutrition, and even death.^2,3^

Previous studies have assessed common measurement inaccuracy of infant formula preparations in relation to bottle characteristics, package instructions, caregiver experience, and target amount, indicating a tendency toward underdilution of formula.^2,4^ The present study addressed accuracy in measurement of powder and water when preparing infant formula in the presence or absence of a simulated crying baby to test the hypothesis that the mean absolute percentage error (MAPE) would be higher with a crying baby present.

## Methods

This cross-sectional study followed the Standards for Quality Improvement Reporting Excellence (SQUIRE) reporting guideline. The study was approved by the institutional review boards at the University of Nevada Las Vegas and Kansas State University. A diverse sample of 84 caregivers from Kansas and Nevada participated. Participants provided signed informed consent and were randomly assigned to begin preparing infant formula with or without the presence of a simulated crying baby (ie, holding life-size doll and hearing crying infant recording). Participants hand-scooped formula powder and poured water to prepare 4 oz and 7 oz feedings, using both their own product and a standardized set of products. Measurement weights were recorded using a calibrated high-precision electronic analytical balance. The weights listed on the formula label served as the theoretical reference standard for calculating MAPE, log-transformed due to positive skew. Linear mixed effects models were used to estimate the primary fixed effects of crying baby presence or absence, controlling for target amount (4 oz vs 7 oz), and products (participant vs researcher) on MAPE measurement. Data were analyzed from July to August 2024. Data were analyzed using R version 4.0.3 (R Project for Statistical Computing). Statistical significance was set at *P* < .05, and tests were 2-sided.

## Results

The 84 caregivers had a mean (SD) age of 31.1 (7.4) years (Table). The Figure displays resultant distributions of percentage error for powder, water, and combined powder and water measurements. Descriptively, MAPE was 13.9% (95% CI, 10.8%-17.1%) for the combination of powder and water with a crying baby present and 13.0% (95% CI, 11.0%-15.1%) without the baby. Approximately one-third of powder and water combinations (119 of 334 with a baby present [35.7%] and 115 of 336 without a baby present [34.2%]) had at least 10% MAPE (large errors). The presence of the simulated crying baby was associated with no significant difference in formula powder MAPE (10.0% [95% CI, 8.5%-11.4%] vs 9.0% [95% CI, 7.8%-10.3%]; β for baby, 1.16 [95% CI, 0.99-1.34]; *P* = .06), suggesting 16% more error with a crying baby present. For water, MAPE was similar regardless of baby presence (4.4% [95% CI, 3.5%-5.4%] vs 4.3% [95% CI, 3.4%-5.3%]; β for baby, 0.96 [95% CI, 0.80-1.14]; *P* = .63). Generalized linear mixed models analyzing the odds of large errors for the combination of powder and water showed no significant difference with or without a crying baby present.

**Table.** Participant Demographics and Characteristics (N = 84)

Caregiver characteristic	Subgroup, No. (% of sample)
Age, mean (SD), y^a^	31.1 (7.4)
Infant’s age, mean (SD), mo^b^	7.2 (5.2)
Main caregiver	
Yes	71 (84.5)
No	12 (14.3)
Not reported	1 (1.2)
WIC food security program participation	
Yes	32 (38.1)
No	51 (60.7)
Not reported	1 (1.2)
Currently smoking or vaping	
Yes	16 (19.0)
No	67 (79.8)
Not reported	1 (1.2)
Currently also breastfeeding	
Yes	30 (35.7)
No	54 (64.3)
Not reported	0
Hispanic	
Yes	39 (46.4)
No	44 (52.4)
Not reported	1 (1.2)
Race	
American Indian or Alaska Native	1 (1.2)
Asian	4 (4.8)
Black or African American	7 (8.3)
Mixed or other race	27 (32.1)
White	44 (52.4)
Not reported	2 (2.4)
Education	
Less than high school graduate	5 (6.0)
High school graduate	19 (22.6)
Some college or associate’s degree	31 (36.9)
Bachelor’s degree or higher	29 (34.5)
Not reported	1 (1.2)
Household income, $	
<35 000	22 (26.2)
35 000 to <100 000	38 (45.2)
≥100 000	21 (25.0)
Not reported	3 (3.6)
Breastfeeding duration, mo	
Never or <1	39 (46.4)
1 to 5	27 (32.1)
≥6	18 (21.4)
Not reported	0
Frequency of bottle feeding, times daily	
1 to 3	29 (34.5)
≥4	54 (64.3)
Not reported	1 (1.2)
Amount of formula used, oz per feeding	
≤2	5 (6.0)
3 to 5	42 (50.0)
≥6 oz	35 (41.7)
Not reported	3 (3.6)

Abbreviation: WIC, Women, Infant, and Children.

^a^ The caregiver age was not reported for 5 (6.0%) of the sample.

^b^ The infant age was not reported for 1 (1.2%) of the sample.

**Figure.**
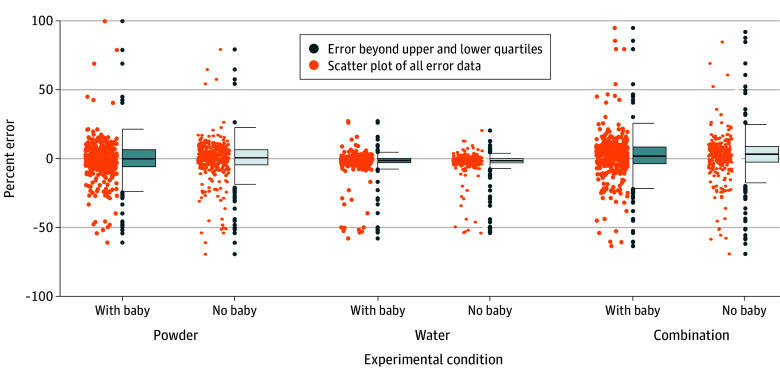
Mean Percent Error for Infant Formula Powder, Water, and Combination of Powder and Water Measurement Distributions by Crying Baby Presence or Absence The y-axis represents the percentage error, while the x-axis displays the experimental conditions: powder, water, and combination. Each were split into 2 categories: with baby and no baby. The dots indicate individual estimated percentage errors for each condition, while the box plots represent the median (IQR) of the percentage error distribution.

## Discussion

Our findings revealed that caregivers frequently made large measurement errors when preparing infant formula, with no significant difference in error during presence of a simulated crying baby. The small change in measurement error due to the crying baby only marginally affected accuracy. The mean size of measurement errors equates to approximately 10 kcal per 4 oz feeding or 18 kcal per 7 oz feeding, similar to previous studies with a tendency toward underdilution.^4,5,6^ Our results showed greater measurement error was observed from scooping powder than from pouring water but those errors compounded for the combination of powder and water. Due to possible negative health outcomes from overdilution and underdilution errors, some caregivers and infants may benefit from interventions to improve formula-feeding practices. This study was limited by convenience sampling and the laboratory-based setting, which used a simulated crying infant. Future studies in this area should explore additional factors, such as sleep deprivation and social determinants of health, that may contribute to measurement errors and develop targeted strategies to enhance caregiver precision in formula preparation.

## Notes

Supplement.
Data Sharing Statement
