# Supplementary material for: Accuracy in Measurement of Infant Formula Powder and Water by Caregivers With and Without a Crying Baby Present
Source: JAMA Netw Open. 2024 Dec 4;7(12):e2447362. doi: 10.1001/jamanetworkopen.2024.47362 (PMC11618465; doi:10.1001/jamanetworkopen.2024.47362)
Supplement: Supplement. — Data Sharing Statement [file jamanetwopen-e2447362-s001.pdf]

# Data Sharing Statement

Rosenkranz. Accuracy in Measurement of Infant Formula Powder and Water by Caregivers With and Without a Crying Baby Present. *JAMA Netw Open*. Published December 04, 2024. doi:10.1001/jamanetworkopen.2024.47362

**Data**
**Data available:** Yes
**Data types:** Deidentified participant data
**How to access data:** Richard.rosenkranz@unlv.edu
**When available:** With publication

**Supporting Documents**
**Document types:** None

**Additional Information**
**Who can access the data:** Researchers whose proposed use of the data has been approved
**Types of analyses:** For any purpose
**Mechanisms of data availability:** Without investigator support
